# The Pattern of mRNA Expression Is Changed in Sinoatrial Node from Goto-Kakizaki Type 2 Diabetic Rat Heart

**DOI:** 10.1155/2018/8454078

**Published:** 2018-09-02

**Authors:** F. C. Howarth, M. A. Qureshi, P. Jayaprakash, K. Parekh, M. Oz, H. Dobrzynski, T. E. Adrian

**Affiliations:** ^1^Department of Physiology, College of Medicine & Health Sciences, UAE University, Al Ain, UAE; ^2^Department of Pharmacology, College of Medicine & Health Sciences, UAE University, Al Ain, UAE; ^3^Cardiovascular Sciences, University of Manchester, Manchester, UK; ^4^Department of Basic Medical Sciences, Mohammed Bin Rashid University of Medicine & Health Sciences, Dubai, UAE

## Abstract

**Background:**

*In vivo* experiments in Goto-Kakizaki (GK) type 2 diabetic rats have demonstrated reductions in heart rate from a young age. The expression of genes encoding more than 70 proteins that are associated with the generation and conduction of electrical activity in the GK sinoatrial node (SAN) have been evaluated to further clarify the molecular basis of the low heart rate.

**Materials and Methods:**

Heart rate and expression of genes were evaluated with an extracellular electrode and real-time RT-PCR, respectively. Rats aged 12-13 months were employed in these experiments.

**Results:**

Isolated spontaneous heart rate was reduced in GK heart (161 ± 12 bpm) compared to controls (229 ± 11 bpm). There were many differences in expression of mRNA, and some of these differences were of particular interest. Compared to control SAN, expression of some genes were downregulated in GK-SAN: gap junction, *Gja1* (Cx43), *Gja5* (Cx40), *Gjc1* (Cx45), and *Gjd3* (Cx31.9); cell membrane transport, *Trpc1* (TRPC1) and Trpc6 (TRPC6); hyperpolarization-activated cyclic nucleotide-gated channels, *Hcn1* (HCN1) and *Hcn4* (HCN4); calcium channels, *Cacna1d* (Ca_v_1.3), *Cacna1g* (Ca_v_3.1), *Cacna1h* (Ca_v_3.2), *Cacna2d1* (Ca_v_*α*2*δ*1), *Cacna2d3* (Ca_v_*α*2*δ*3), and *Cacng4* (Cav*_γ_*4); and potassium channels, *Kcna2* (K_v_1.2), *Kcna4* (K_v_1.4), *Kcna5* (K_v_1.5), *Kcnb1 (*K_v_2.1), *Kcnd3* (K_v_4.3), *Kcnj2* (K_ir_2.1), *Kcnk1* (TWIK1), *Kcnk5* (K_2P_5.1), *Kcnk6* (TWIK2), and *Kcnn2* (SK2) whilst others were upregulated in GK-SAN: *Ryr2* (RYR2) and *Nppb* (BNP).

**Conclusions:**

This study provides new insight into the changing expression of genes in the sinoatrial node of diabetic heart.

## 1. Introduction

Cardiovascular complications are widely reported in diabetic patients and may be associated with various cardiac arrhythmias and sudden cardiac death [1–5]. Although coronary artery disease and hypertension are risk factors for cardiovascular dysfunction in diabetic patients, there is also a risk of developing cardiac dysfunction that is independent of coronary atherosclerosis and hypertension [6]. Electrical disturbances have been widely reported in diabetic heart [7, 8]. Bolognesi et al. [9] reported that sinus bradycardia and QT prolongation can occur in insulin-treated diabetic patients with severe hypoglycemia. Abnormal functions of sinus node automaticity, third-degree atrioventricular block, and left bundle branch block occur more frequently in diabetic patients [10–12]. Type 2 diabetic patients have an increased risk of supraventricular arrhythmias including atrial fibrillation [1, 13–16], ventricular tachyarrhythmias, and ventricular fibrillation [3, 5, 7, 17]. Various studies have shown that QT prolongation is an independent risk factor for cardiovascular mortality in diabetic patients [2, 18–21]. Howarth et al. [22] reported disturbances in the electrocardiogram including bradycardia and prolongation of the QRS and QT intervals in the GK rat. Soltysinska et al. [23] reported alterations in systolic and diastolic function and prolonged SAN recovery time in db/db diabetic mice. Hyperglycemia, a hallmark of diabetes mellitus, is associated with oxidative stress which in turn exacerbates inflammation and further exacerbates oxidative stress, which in turn may partly underlie QT prolongation and trigger ventricular arrhythmias [24, 25].

In the Zucker diabetic fatty rat, myocardial impulse propagation was impaired [26]. Little is known about the effects of type 2 diabetes mellitus (T2DM) on the electrophysiology of the SAN. In the streptozotocin- (STZ-) induced diabetic rat, SAN conduction, pacemaker cycle length, and action potential duration were prolonged [27, 28]. Various ion channels and ionic conductances including L-type and T-type Ca^2+^ current, hyperpolarization-activated “funny” current, Na^+^ current, Na^+^/Ca^2+^ exchange current, and various K^+^ currents are essential for the generation, propagation, and regulation of the SAN action potential [29]. Sarcoplasmic reticulum (SR) Ca^2+^ might also contribute to the generation and decay of the SAN action potential [30]. Structural and/or functional channelopathies may underlie some of the electrical abnormalities that have been reported in diabetic heart [22]. In order to further elucidate the molecular basis of these heart rhythm disturbances, we have investigated the pattern of more than 70 genes encoding proteins that are associated with the generation and conduction of electrical activity in the SAN in the GK type 2 diabetic heart. Results from this study will provide direction for future structural and functional studies of the electrical conduction system in the diabetic SAN.

## 2. Materials and Methods

### 2.1. Experimental Protocol

Ethical approval for this project was obtained from the Animal Ethics Committee, College of Medicine & Health Sciences, UAE University. Male GK and Wistar control rats were reared as previously described [31]. Rats were kept in cages, under a 12 h-12 h light-dark cycle, and had free access to food and tap water. Room temperature was kept between 21 and 25°C. Experiments commenced when the animals were 12–13 months of age. Blood glucose, after an overnight fast, and blood glucose 120 min after a glucose challenge (2 g/kg body weight, intraperitoneal) were measured in GK and age-matched controls. Prior to experiments, the body weight, heart weight, and the nonfasting blood glucose were also measured. The heart to body weight ratio was calculated.

### 2.2. Measurement of Heart Rate

Rats were sacrificed as previously described using a guillotine [32]. The chest was then opened, and the hearts were rapidly removed and mounted in Langendorff mode and perfused at a constant flow rate of 8 ml.g heart^−1^ min^−1^ at 36–37°C with normal Tyrode containing 140 mM NaCl, 5 mM KCl, 1 mM MgCl_2_, 10 mM glucose, 5 mM HEPES, and 1.8 mM CaCl_2_ and adjusted to pH 7.4 with NaOH bubbled with oxygen. An extracellular suction electrode was used to measure heart rate according to previously described techniques [33]. Action potentials were recorded in the left ventricle. Electrical signals were collected at 400 Hz. Signals were then amplified (ADInstuments, ML136 Bioamp), delivered to a Powerlab (ADInstruments, PL410), and displayed on a PC monitor. Analysis was performed using ADInstruments software version v 4.21 (ADInstruments, Australia).

### 2.3. Expression of mRNA

Previously described techniques were used to evaluate the expression of genes encoding more than 70 proteins involved in electrical activity in the SAN [32, 34–36]. After rats were sacrificed, the hearts were rapidly removed and placed in a plastic dish containing NaCl 140 mM, KCl 5.4 mM, MgCl_2_ 1 mM, HEPES 5 mM, D-glucose 5.5 mM, and CaCl_2_ 1.8 mM and adjusted to pH 7.4 with NaOH. The ventricles and the left atrium were removed, and the right atrium was opened to expose the SAN and crista terminalis. The SAN artery was used to identify the SAN. The SAN was exposed, and 2 mm biopsy samples of SAN were carefully collected from GK and control rat hearts according to previously described techniques [32]. The samples were placed in RNAlater (AM7021, Life Technologies, Carlsbad, CA, USA) and stored overnight at room temperature to allow thorough penetration of the tissue [32]. The following day, tissue samples were frozen at −20°C in readiness for further processing. Tissue samples were homogenized in homogenization microtubes containing 1.4 mm ceramic beads using a Precellys 24 tissue homogenizer (Bertin Technologies, USA). The homogenization protocol comprised 2 runs at 6500 rpm of 20 seconds each with a 15-second gap. The SV Total RNA Isolation System (Promega, Madison, USA) was used to isolate total RNA from the tissue, in accordance with the manufacturer's instructions. Spectrophotometric techniques were used to measure the concentration and purity of the RNA samples. The absorbance was measured at 260 nm, and the ratio of absorbance was measured at 260 nm and 280 nm (ND-1000, NanoDrop). cDNA was generated using a 2-step RT-PCR procedure. Total RNA (500 ng) was converted into cDNA in a 25 *μ*l PCR reaction with 10x RT Buffer 2.0 *μ*l, 25x dNTP Mix (100 mM) 0.8 *μ*l, 10x RT Random Primers 2.0 *μ*l, MultiScribe™ Reverse Transcriptase 1.0 *μ*l, RNase inhibitor 1.0 *μ*l, and Nuclease-free H_2_O (High Capacity cDNA Reverse Transcription Kit, 4374966, Applied Biosystems, USA). A Veriti thermal cycler (Applied Biosystems, USA) was used to perform reverse transcription. The cycles were as follows: 10 min at 5°C, 120 min at 37°C, and 5 min at 85°C. Customized TaqMan Low Density Arrays (Format 32, 4346799, Applied Biosystems, USA) were used for gene expression assays. The TaqMan assays were preloaded in triplicate for each RNA sample. Rat glyceraldehyde-3-phosphate dehydrogenase (GAPDH) was selected as the endogenous control [35, 37]. The expression of GAPDH was not significantly different (*p* > 0.05) between GK and control SAN samples. 100 ng of cDNA was loaded, together with 2x TaqMan Gene Expression Master Mix (No AmpErase UNG, Applied Biosystems, USA), for a total of 100 *μ*l per port. Two SAN tissue samples were combined for each real-time RT-PCR assay. Real-time RT-PCR was carried out in a Fast ABI Prism 7900HT Sequence Detection System (Applied Biosystems, USA). Standard mode PCR thermal cycling parameters were run as follows: 2 min at 50°C, 10 min at 94.5°C, and 40 cycles of 30 sec at 97°C and 59.7°C for 1 min. Analysis was performed using ABI Prism 7900HT SDS, v2.4 software. Statistical analysis was performed using SDS RQ Manager 1.1.4 software employing the 2^−ΔΔCt^ method with a relative quantification RQmin/RQmax confidence set at 95%. The lists of target genes, encoded proteins, and description of the proteins are shown in Table 1.

### 2.4. Statistics

Data were expressed as the mean ± SEM of “*n*” observations. Statistical comparisons were performed using an independent samples *t*-test (SPSS v. 20), and a “*p*” value of less than 0.5 was considered to indicate a significant difference.

## 3. Results

### 3.1. General Characteristics

Nonfasting blood glucose and fasting blood glucose were significantly (*p* < 0.01) elevated in GK rats compared to age-matched controls. After an overnight fast blood glucose, 120 min following a glucose challenge (2 g/kg body weight, intraperitoneal), was also significantly (*p* < 0.01) elevated in GK compared to controls. Body weight and heart weight were significantly (*p* < 0.01) increased, and heart weight/body weight ratio was significantly (*p* < 0.05) reduced in GK compared to controls (Table 2).

### 3.2. Heart Rate

**Figure 1:**
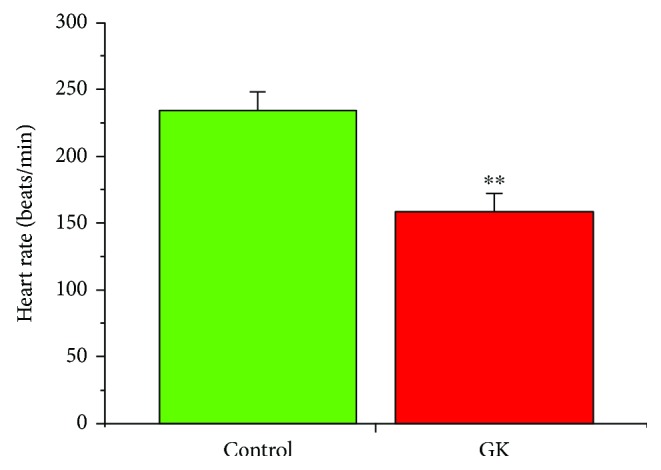
Heart rates in GK and control rats. Data are mean ± SEM, *n* = 10 hearts.

Heart rate is shown in Figure 1. Heart rate was significantly (*p* < 0.01) reduced in GK (161 ± 12 bpm, *n* = 10) compared to control (229 ± 11 bpm, *n* = 10) heart (Figure 1).

### 3.3. Expression of mRNA

**Figure 2:**
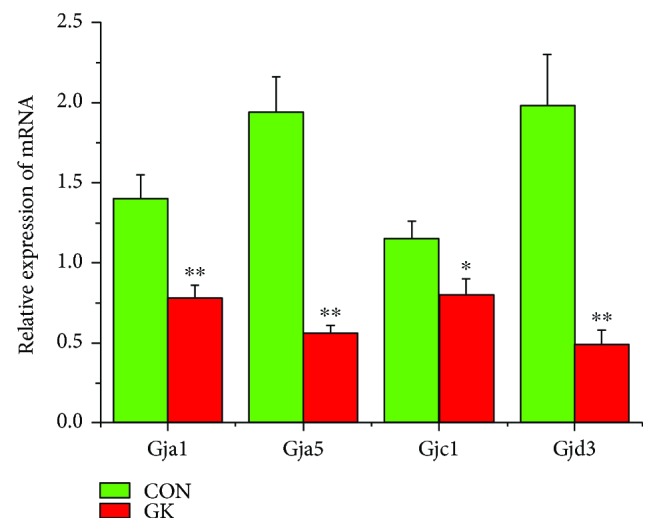
Expression of genes encoding various gap junction proteins. Data are mean ± SEM, *n* = 9 samples, each containing SANs from 2 hearts.

**Figure 3:**
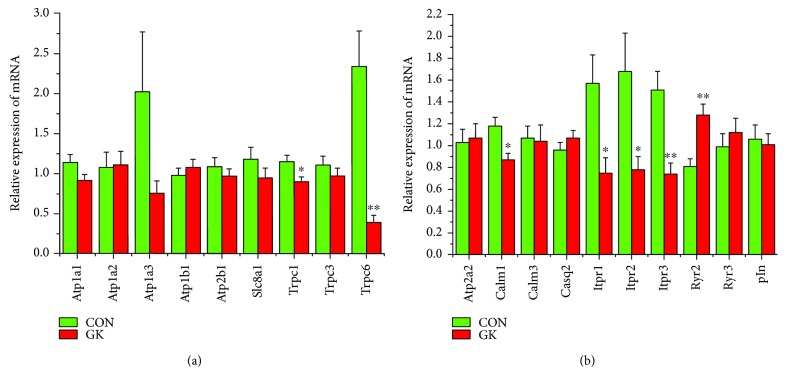
(a) Expression of genes encoding various membrane transport and (b) intracellular Ca^2+^ transport and regulatory proteins. Data are mean ± SEM, *n* = 6–9 samples, each containing SANs from 2 hearts.

**Figure 4:**
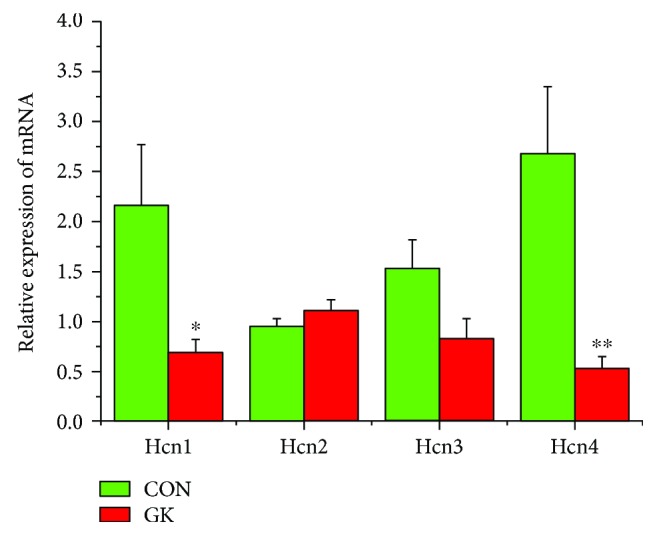
Expression of genes encoding various hyperpolarization-activated cyclic nucleotide-gated channels. Data are mean ± SEM, *n* = 6–8 samples, each containing SANs from 2 hearts.

**Figure 5:**
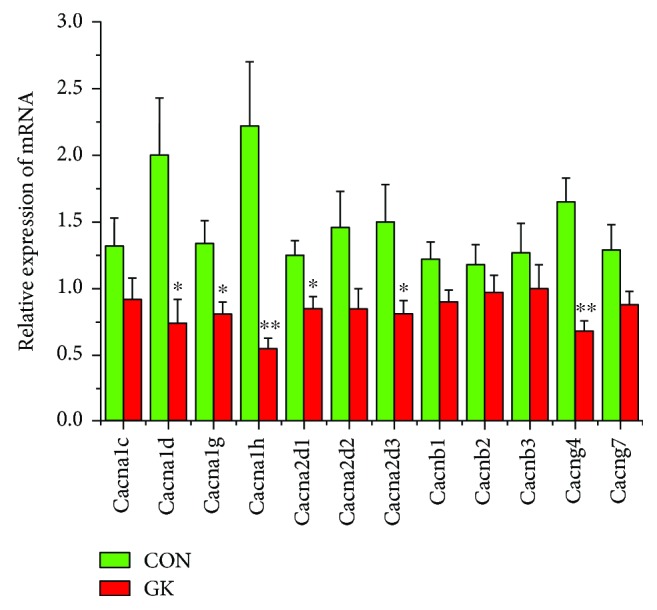
Expression of genes encoding various calcium channel proteins. Data are mean ± SEM, *n* = 7–9 samples, each containing SANs from 2 hearts.

**Figure 6:**
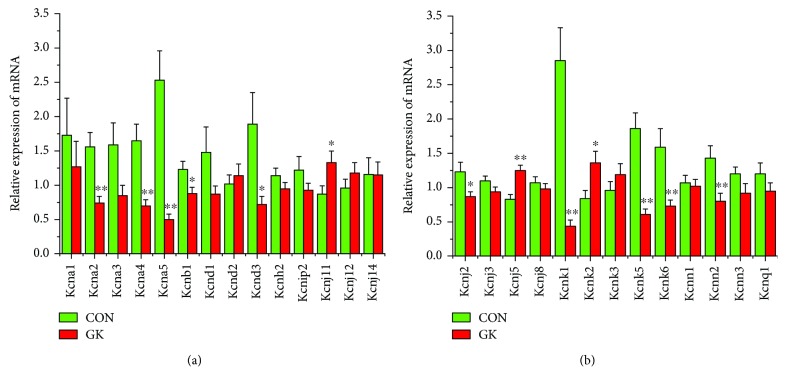
(a) and (b) Expression of genes encoding various potassium channel proteins. Data are mean ± SEM, *n* = 8–9 samples, each containing SANs from 2 hearts.

**Figure 7:**
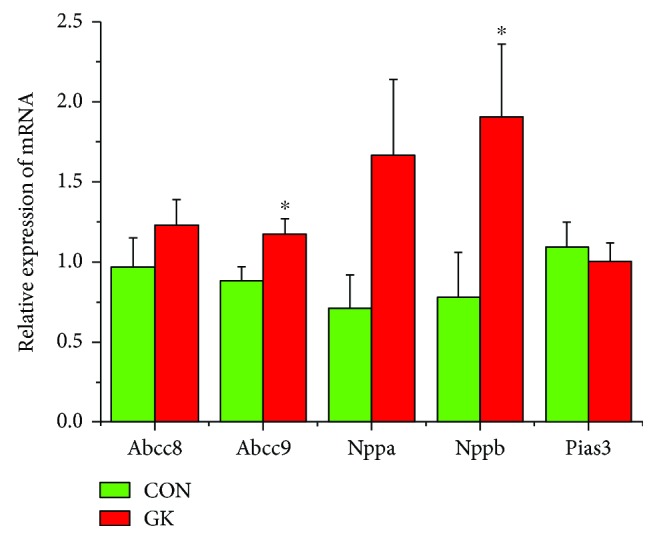
Expression of genes encoding miscellaneous cardiac proteins. Data are mean ± SEM, *n* = 5–9 samples, each containing SANs from 2 hearts.

Figure 2 shows the expression of mRNA for gap junction proteins. Expression of *Gja1* (2-fold), *Gja5* (4-fold), *Gjc1*, and *Gjd3* (4-fold) were all downregulated in GK compared to control SAN. Figures 3(a) and 3(b) show expression of mRNA for cell membrane transport and intracellular Ca^2+^ transport and regulatory proteins, respectively. Expression of *Trpc1* and *Trpc6* (6-fold) and *Itpr1*–*3* (2-fold) were downregulated, and *Ryr2* was upregulated in GK compared to control SAN.  Figure 4 shows the expression of mRNA for hyperpolarization-activated cyclic nucleotide-gated channel proteins. Expression of *Hcn1* (4-fold) and *Hcn4* (5-fold) were downregulated in GK compared to control SAN.  Figure 5 shows the expression of mRNA for calcium channel proteins. Expression of *Cacna1d* (3-fold), *Cacna1g*, *Cacna1h* (4-fold), *Cacna2d1*, *Cacna2d3*, and *Cacng4* (3-fold) were downregulated in GK compared to control SAN. Figures 6(a) and 6(b) show the expression of mRNA for potassium channel proteins. Expression of *Kcna2*, *Kcna4*, *Kcna5* (5-fold), *Kcnb1*, *Kcnd3*, *Kcnj2*, *Kcnk1* (7-fold), *Kcnk5* (3-fold), *Kcnk6*, and *Kcnn2* were downregulated, and *Kcnj11*, *Kcnj5*, and *Kcnk2* were upregulated in GK compared to control SAN.  Figure 7 shows the expression of mRNA for various other proteins that might affect electrical activity in the SAN. Expression of *Abcc9* and *Nppb* were upregulated in GK compared to control SAN.

## 4. Discussion

Previous *in vivo* biotelemetry experiments performed in GK type 2 diabetic rats have demonstrated disturbances in the electrocardiogram. These disturbances have included reduced heart rate which was associated with prolonged QRS and QT intervals [22]. The spontaneous heart beat in isolated perfused heart was also lower in GK compared to control rats which suggests that the bradycardia is at least partly attributed to an intrinsic defect in the electrical conduction system of the heart [33]. Changes in membrane potential that occur during the different phases of the SAN action potential are produced by changes in the movement of various ions (Na^+^, Ca^2+^, and K^+^) across the cell membrane and by the movement of Ca^2+^ in or out of the SR [30].

Disturbances in one or more of these ionic conductances would be expected to alter the electrophysiological properties of the SAN. In order to further clarify the molecular basis of the low heart rate in the GK rat, the expression of more than 70 genes that encode proteins that are associated with the generation and conduction of electrical activity in the GK SAN were evaluated.

Regarding the changes in mRNA of particular interest were (i) downregulation of *Gja5* and *Gjd3* (4-fold), (ii) downregulation of *Trpc6* (6-fold), (iii) downregulation of *Hcn4* (5-fold), (iv) downregulation of *Cacna1d* (3-fold), *Cacna1h* (4-fold), and *Cacng4* (3-fold), (v) downregulation of *Kcna5* (5-fold) and *Kcnk1* (7-fold) in GK compared to control SAN, and (vi) upregulation of *Nppb* in GK compared to control SAN.

*Gja5* (Cx40) and *Gjd3* (Cx31.9) were downregulated in GK compared to control SAN. The connexins are structurally related proteins that assemble to form gap junctions which play an important role in cell-to-cell electrical communication and cardiac rhythmicity. At least 5 connexins (Connexin30.2, Connexin37, Connexin40, Connexin43, and Connexin45) are expressed in the heart, and each connexin displays regional and cell type-specific expression [38]. Downregulation of connexin proteins may result in impaired electrical conduction between cells and, hence, may have implications for electrical transmission among the cells of the SAN and between the SAN and other regions of the heart [27].

*Trpc6* (TRPC6) and to a lesser extent *Trpc1* (TRPC1) were downregulated in GK compared to control SAN. The transient receptor potential channels (TRPCs) are a large family of ion channels that are widely expressed in human tissue including the heart and the vasculature [39, 40]. TRPC1 and TRPC6 are stretch-activated, nonselective cation channels expressed in ventricular muscle from mouse heart [41]. The TRPCs have been found to play a role in cardiovascular disease [42]. Upregulation of TRPCs is involved in the pathophysiology of cardiac hypertrophy and heart failure [39, 43, 44]. Previous studies have demonstrated that cardiac hypertrophy upregulated TRPC6 and inhibition of TRPC6 suppressed agonist-induced hypertrophic responses [45–47]. Downregulation of *Trpc1* and *Trpc6* in the GK SAN may have implications for the conduction of Na^+^ or Ca^2+^ current through nonselective TRPCs and in turn implications for the generation of action potentials in SAN cells [43, 48, 49].

The SAN generates action potentials automatically, and the cells of the SAN appear to have two separate but closely communicating mechanisms (often referred to as “clocks”). There is a “membrane clock” which consists of ion channels that include the hyperpolarization-activated cyclic nucleotide-gated channels (mainly HCN4), the L-type Ca^2+^ channels (mainly Cav1.3), and the T-type Ca^2+^ channels (mainly Cav3.1). There is also a “calcium clock” which consists of Ca^2+^-handling proteins that include the ryanodine receptor (RyR2), the sarcoplasmic reticulum-ATPase (SERCA2a), and the Na^+^/Ca^2+^ exchanger (NCX1) [50, 51]. Recent studies in human nodal cells have shown that when the clocks become uncoupled, SAN cells fail to generate spontaneous action potentials and *β*-adrenergic receptor stimulation, which in turn increases cyclic AMP concentration and was able to restore spontaneous, rhythmic action potentials [51]. HCN1–4 proteins are the structural components of the funny channels, and the funny current (I_f_) is the main electrical driving force behind diastolic depolarization.

*Hcn4* (HCN4) and to a lesser extent *Hcn1* (HCN1) were downregulated in GK compared to control SAN. Shinagawa et al. [29] have shown that a variety of time-dependent and voltage-dependent ionic currents contribute to the rat action potential including the delayed rectifier K^+^ current, the L-type Ca^2+^ current, and Na^+^ current. *Hcn4* and *Hcn1* are variously expressed in rat atrioventricular node, SAN, right atrium, and ventricle [52]. In human, SAN *Hcn4* accounts for 75%, *Hcn1* for 21%, *Hcn2* for 3%, and *Hcn3* for 0.7% of the *Hcn* in human SAN [53]. It has been shown that *Hcn4*-related inheritable arrhythmias, resulting in four different mutations of the *Hcn4* gene, are associated with reduced heart rate and various arrhythmias [54]. *Hcn1* (HCN1) was reduced in GK compared to control SAN. In the absence of the pacemaker channel protein HCN1, mice display congenital SAN dysfunction that was characterized by low heart rate, sinus arrhythmia, increased SAN conduction and recovery time, and recurrent sinus pauses [55]. Downregulation of *Hcn1* and *Hcn4* might be expected to reduce the slope of the pacemaker potential, prolong the time to threshold potential, and partly underlie the bradycardia in the GK rat heart.

*Cacna1d* (Ca_v_1.3), *Cacna1h* (*Ca_v_3.2*), and *Cacng4* (Ca_v_*γ*4) were downregulated in GK compared to control SAN. Voltage-gated L-type and T-type Ca^2+^ channels are found in the heart. L-type and T-type Ca^2+^ currents are important in the generation of phase 4, and L-type Ca^2+^ current is also important in the generation of phase 0 of SAN and atrioventricular node action potentials. The T-type Ca^2+^ channels also have roles in cell growth and cardiovascular remodeling. *Cacna1g* (Ca_v_3.1) and *Cacna1h* (Ca_v_3.2) encode pore-forming alpha(1) subunits of the T-type Ca^2+^ channel in cardiac muscle [56]. *Cacna1g* (Ca_v_3.1) and *Cacna1h* (Ca_v_3.2) may be involved in the development of the heart's electrical conduction system [57]. In adult heart, the T-type Ca^2+^ channel is widely distributed in the conduction system and plays an important role in the generation of the pacemaker depolarization in phase 4 of the SAN action potential [58]. Downregulation of *Cacna1h* (Cav3.2) might have consequences for the generation of the pacemaker potential and, therefore, the heart rate in the GK SAN. Interestingly, *Cacna1c* (Cav1.2) which mediates the generation of the pacemaker potential and action potential in the SAN was also modestly reduced in GK compared to control SAN. The gamma subunits of the L-type Ca^2+^ channel, of which there are 8 isoforms, differentially modulate Ca^2+^ channel function [59]. Reductions in expression of *Cacna1d* (Ca_v_1.3) and *Cacng4* (Cav*γ*4) would be consistent with depressed L-type Ca^2+^ current and modulation of the L-type Ca^2+^ channel activity and have implications for the pacemaker potential and generation of the action potential and, hence, heart rate in the GK rat heart.

Compared to controls, the general characteristics of the GK rat included elevated nonfasting and fasting blood glucose and glucose intolerance, as evidenced by the raised blood glucose 120 min following a glucose challenge, after an overnight fast. RyR2 channels are found in sarcoplasmic reticulum and have important roles in cardiac myocyte contraction and the generation of autorhythmicity in SAN cells [51]. Recent studies have revealed a crucial role of RyR2 channels in the regulation of insulin release and glucose homeostasis [60]. RyR2 was upregulated in GK compared to CON SAN, and it would be interesting to investigate if the expression of pancreatic *β*-cell RyR2 is also altered in GK rat which in turn might partly underlie the hyperinsulinemia previously reported in GK compared to CON rat [61].

*Kcna5* (K_v_1.5) and *Kcnk1* (TWIK1) were downregulated in GK compared to control SAN. *Kcna5* (Kv1.5) encodes the potassium channel *α*-subunit K_v_1.5 and is widely expressed in heart, brain, and vascular, airway, and smooth muscle cells [62–64]. Within the heart, several studies have demonstrated the presence of K_v_1.5 in atria, ventricle, and SAN [65–67]. The cardiac ultrarapid outward (I_Kur_) current, which is encoded by *Kcna5*, controls action potential duration and in particular repolarizing current [68–70]. Remodeling of ion channels, including the voltage-gated potassium channel K_v_1.5, is involved in the pathophysiology of atrial fibrillation [71]. Expression of K_v_1.5 results in marked decreases in mouse ventricular myocyte action potential duration [72]. *In vivo* overexpression of K_v_1.5 has been shown to shorten action potential duration, eliminate after depolarizations, and increase heart rate in mice with long QT syndrome [73]. Downregulation of *Kcna5* might result in prolonged repolarization and duration of the action potential and, as a consequence, reduction in heart rate. *Kcnk1* (TWIK1) is widely expressed in the human heart and brain [74]. TWIK1 channels may be involved in the regulation of the resting membrane potential and, hence, excitability of the cardiac myocyte [75, 76]. Downregulation of *Kcnk1* might have implications for resting membrane potential and, hence, excitability of the SAN cell. Abnormal QT prolongation is not an uncommon feature in diabetic heart, and this is associated with reduction in the rapid delayed rectifier K^+^ current (I_Kr_) in insulin-dependent diabetic heart [77]. *Kcnh2*, the gene that encodes expression of KCNH2 protein (also known as hERG1) that encodes the hERG channel, was not significantly altered in diabetic SAN.

*Nppb* (BNP) and to a lesser extent *Nppa* (ANP) were upregulated in GK compared to control SAN. BNP and ANP are secreted by the atria and ventricles and cause a reduction in blood pressure and cardiac hypertrophy. In the heart, BNP reduces ventricular fibrosis, and BNP and ANP are involved in the pathophysiology of heart failure, coronary heart disease, hypertension, and left ventricular hypertrophy [78–80]. Increases in BNP and ANP in blood plasma and atrial tissues are associated with varying effects of these natriuretic peptides on the amplitude and kinetics of shortening and intracellular Ca^2+^ in ventricular myocytes from STZ-induced diabetic rat [81, 82]. Springer et al. [83] reported that BNP increased electrical conduction velocity and heart rate in isolated heart and in the SAN and also increased spontaneous action potential frequency in SAN cells. Upregulation of *Nppb* (BNP) and *Nppa* (ANP) may be associated with mechanisms that compensate for low heart rate in the GK type 2 diabetic rat heart.

It is of interest to compare gene expression in the current study with that in a recent study in SAN from the STZ-induced diabetic rat [32]. For example, *Gja5* (Cx40) and *Gjd3* (Cx31.9) were downregulated in GK and unaltered in STZ SAN; *Trpc6* (TRPC6) was downregulated in GK and upregulated in STZ SAN; *Ryr2* (RYR2) was upregulated in GK and unaltered in STZ SAN; *Hcn1* (HCN1) and *Hcn4* (HCN4) were downregulated in GK and unaltered in STZ SAN; *Cacna1d* (Ca_v_1.3), *Cacna1h* (Ca_v_3.2), and *Cacng4* (Ca_v_*γ*4) were downregulated in GK SAN; however, in the STZ study, *Cacna1d* was unaltered, *Cacna1h* was upregulated, and *Cacng4* was downregulated; *Kcna5* (K_v_1.5) and *Kcnk1* (TWIK1) were downregulated in GK SAN and unaltered in STZ SAN; *Nppb* (BNP) was upregulated in GK and in STZ SAN. These differences may be partly attributable to the different ages of the animals (GK/CON rats 12–13 months vs. STZ/CON rats 18 weeks of age) and the nature of diabetes (GK genetic vs. STZ chemical induced) in the two experimental models.

Heart failure and T2DM are two increasingly common and related diseases. MicroRNAs (miRs) play an important role in the pathogenesis of structural alterations in the failing heart [84]. Some patients, who are affected by heart failure and who have severe hemodynamic and electrical dysfunction, receive cardiac resynchronization therapy (CRT). Recent studies have shown that CRT is associated with alterations in expression of genes and miRs which regulate a variety of cardiac processes including cardiac apoptosis, cardiac fibrosis, cardiac hypertrophy, cardiac angiogenesis, and membrane channel ionic currents [85, 86]. In the future, interventions that are able to alter expression of miRs might become an important treatment modality for diabetes and heart failure.

The molecular biology results are based on quantitative PCR. The possibility of posttranscriptional modifications, for example, by miR, means that changes in gene expression might not necessarily result in corresponding changes in the expression of proteins. Previous studies have demonstrated that spironolactone regulates HCN protein expression through miR-1 in rats with myocardial infarction and interestingly, upregulation of miR-1 expression partially contributed to the posttranscriptional repression of HCN protein expression [87]. Recent studies have demonstrated that multiple miRs are involved in the regulation of SCN5A/Nav1.5 channel and its *β*1/SCN1B subunit which is responsible for the fast-activating, fast-inactivating sodium current in atrial and ventricular action potentials [88]. Further structural experiments will be required to investigate the expression of selected proteins and the consequences of these structural changes on the electrophysiology of ion channels in the diabetic heart.

It is hoped that results emerging from laboratory studies will eventually translate into treatment modalities, which might include manipulation of miRs, mRNAs, and associated conduction proteins, in order to normalize electrical dysfunction in the diabetic heart.

## 5. Conclusions

In conclusion, this study provides valuable insight into the differences in expression of genes that encode proteins that are involved in the generation and conduction of electrical activity in the SAN in the GK diabetic rat and will form the basis for future structural and electrophysiological studies.

## Figures and Tables

**Table 1:** Target genes, encoded proteins, and description of proteins.

Genes	Proteins	Protein descriptions
Gap junction proteins		
*Gja1*	Cx43	Connexin43
*Gja5*	Cx40	Connexin40
*Gjc1*	Cx45	Connexin45
*Gjd3*	Cx31.9	Connexin31.9
Cell membrane transport		
*Atp1a1*	Na/K ATPase, *α*1	ATPase, Na^+^/K^+^ transporting, alpha 1 polypeptide
*Atp1a2*	Na/K ATPase, *α*2	ATPase, Na^+^/K^+^ transporting, alpha 2 polypeptide
*Atp1a3*	Na/K ATPase, *α*3	ATPase, Na^+^/K^+^ transporting, alpha 3 polypeptide
*Atp1b1*	Na/K ATPase, *β*1	ATPase, Na^+^/K^+^ transporting, beta 1 polypeptide
*Atp2b1*	Na/K ATPase, *β*2	ATPase, Ca^++^ transporting, plasma membrane 1
*Slc8a1*	NCX1	Solute carrier family 8 (sodium/calcium exchanger), member 1
*Trpc1*	TRPC1	Transient receptor potential channel 1
*Trpc3*	TRPC3	Transient receptor potential channel 3
*Trpc6*	TRPC6	Transient receptor potential channel 6
Intercellular Ca^2+^ transport and Ca^2+^ regulation		
*Atp2a2*	SERCA2	Sarcoplasmic/endoplasmic reticulum calcium ATPase 2
*Calm1*	Calm1	Calmodulin 1
*Calm3*	Calm3	Calmodulin 3
*Casq2*	Casq2	Calsequestrin 2
*Itpr1*	IP3R1	Inositol 1,4,5-trisphosphate receptor, type 1
*Itpr2*	IP3R2	Inositol 1,4,5-trisphosphate receptor, type 2
*Itpr3*	IP3R3	Inositol 1,4,5-trisphosphate receptor, type 3
*Pln*	PLB	Phospholamban
*Ryr2*/RYR2	RYR2	Ryanodine receptor 2
*Ryr3*/RYR3	RYR3	Ryanodine receptor 3
Hyperpolarization-activated cyclic nucleotide-gated channels		
*Hcn1*	HCN1	Hyperpolarization-activated cyclic nucleotide-gated channel 1
*Hcn2*	HCN2	Hyperpolarization-activated cyclic nucleotide-gated channel 2
*Hcn3*	HCN3	Hyperpolarization-activated cyclic nucleotide-gated channel 3
*Hcn4*	HCN4	Hyperpolarization-activated cyclic nucleotide-gated channel 4
Calcium channels		
*Cacna1c*	Ca_v_1.2	Voltage-dependent, L-type, alpha 1C subunit
*Cacna1d*	Ca_v_1.3	Voltage-dependent, L-type, alpha 1D subunit
*Cacna1g*	Ca_v_3.1	Voltage-dependent, T-type, alpha 1G subunit
*Cacna1h*	Ca_v_3.2	Voltage-dependent, T-type, alpha 1H subunit
*Cacna2d1*	Ca_v_*α*2δ1	Voltage-dependent, alpha 2/delta subunit 1
*Cacna2d2*	Ca_v_*α*2*δ*2	Voltage-dependent, alpha 2/delta subunit 2
*Cacna2d3*	Ca_v_*α*2*δ*3	Voltage-dependent, alpha 2/delta subunit 3
*Cacnb1*	Ca_v_*β*1	Voltage-dependent, beta 1 subunit
*Cacnb2*	Ca_v_*β*2	Voltage-dependent, beta 2 subunit
*Cacnb3*	Ca_v_*β*3	Voltage-dependent, beta 3 subunit
*Cacng4*	Ca_v_*γ*4	Voltage-dependent, gamma subunit 4
*Cacng7*	Ca_v_*γ*7	Voltage-dependent, gamma subunit 7
Potassium channels		
*Kcna1*	K_v_1.1	Voltage-gated shaker-related subfamily A, member 1
*Kcna2*	K_v_1.2	Voltage-gated shaker-related subfamily A, member 2
*Kcna3*	K_v_1.3	Voltage-gated shaker-related subfamily A, member 3
*Kcna4*	K_v_1.4	Voltage-gated shaker-related subfamily A, member 4
*Kcna5*	K_v_1.5	Voltage-gated shaker-related subfamily A, member 5
*Kcnb1*	K_v_2.1	Voltage-gated shab-related subfamily B, member 1
*Kcnd1*	K_v_4.1	Voltage-gated shal-related subfamily D, member 1
*Kcnd2*	K_v_4.2	Voltage-gated shal-related subfamily D, member 2
*Kcnd3*	K_v_4.3	Voltage-gated shal-related subfamily D, member 3
*Kcnh2*	ERG-1	Ether-A-go-go-related protein 1
*Kcnip2*	KChIP2	Kv channel interacting protein 2
*Kcnj11*	K_ir_6.2	Inwardly rectifying subfamily J, member 11
*Kcnj12*	K_ir_2.2	Inwardly rectifying subfamily J, member 12
*Kcnj14*	K_ir_2.4	Inwardly rectifying subfamily J, member 14
*Kcnj2*	K_ir_2.1	Inwardly rectifying subfamily J, member 2
*Kcnj3*	K_ir_3.1	Inwardly rectifying subfamily J, member 3
*Kcnj5*	K_ir_3.4	Inwardly rectifying subfamily J, member 5
*Kcnj8*	K_ir_6.1	Inwardly rectifying subfamily J, member 8
*Kcnk1*	TWIK1	Two pore domain subfamily K, member 1
*Kcnk2*	TREK1	Two pore domain subfamily K, member 2
*Kcnk3*	K_2P_3.1	Two pore domain subfamily K, member 3
*Kcnk5*	K_2P_5.1	Two pore domain subfamily K, member 5
*Kcnk6*	TWIK2	Two pore domain subfamily K, member 6
*Kcnn1*	SK1	Calcium-activated intermediate/small conductance subfamily N alpha, member 1
*Kcnn2*	SK2	Calcium-activated intermediate/small conductance subfamily N alpha, member 2
*Kcnn3*	SK3	Calcium-activated intermediate/small conductance subfamily N alpha, member 3
*Kcnq1*	K_v_7.1	Voltage-gated KQT-like subfamily Q, member 1
Miscellaneous proteins		
*Abcc8*	SUR1	ATP-binding cassette transporter subfamily C member 8
*Abcc9*	SUR2	ATP-binding cassette subfamily C member 9
*Nppa*	ANP	Atrial natriuretic peptide
*Nppb*	BNP	Brain natriuretic peptide
*Pias3*	KChAP	Protein inhibitor of activated STAT3

**Table 2:** General characteristics of GK rats.

	Control	GK	*p* value
Nonfasting blood glucose (mg/dl)	93.00 ± 3.28	128.50 ± 5.06^∗∗^	0.001
Fasting blood glucose (mg/dl)	60.60 ± 1.99	108.75 ± 4.99^∗∗^	0.001
Blood glucose 120 min post glucose challenge (mg/dl)	97.10 ± 2.14	230.55 ± 12.69^∗∗^	0.001
Body weight (g)	369.10 ± 7.03	414.90 ± 7.10^∗∗^	0.001
Heart weight (g)	1.26 ± 0.02	1.37 ± 0.02^∗∗^	0.001
Heart weight/body weight ratio	3.43 ± 0.04	3.30 ± 0.05^∗^	0.049

Data are mean ± SEM, *n* = 10 hearts, ^∗∗^*p* < 0.01, ^∗^*p* < 0.05.

## Data Availability

The raw mRNA data and statistically analyzed data presented in the figures are available, should it be requested, or available from the corresponding author upon request.
